# NMDA Receptors Contribute to Retrograde Synaptic Transmission from Ganglion Cell Photoreceptors to Dopaminergic Amacrine Cells

**DOI:** 10.3389/fncel.2017.00279

**Published:** 2017-09-14

**Authors:** Lei-Lei Liu, Nathan J. Spix, Dao-Qi Zhang

**Affiliations:** Eye Research Institute, Oakland University Rochester, MI, United States

**Keywords:** dopamine, melanopsin, NMDA receptor, amacrine cell, retina

## Abstract

Recently, a line of evidence has demonstrated that the vertebrate retina possesses a novel retrograde signaling pathway. In this pathway, phototransduction is initiated by the photopigment melanopsin, which is expressed in a small population of retinal ganglion cells. These ganglion cell photoreceptors then signal to dopaminergic amacrine cells (DACs) through glutamatergic synapses, influencing visual light adaptation. We have previously demonstrated that in Mg^2+^-containing solution, α-amino-3-hydroxyl-5-methyl-4-isoxazole-propionate (AMPA) receptors mediate this glutamatergic transmission. Here, we demonstrate that removing extracellular Mg^2+^ enhances melanopsin-based DAC light responses at membrane potentials more negative than −40 mV. Melanopsin-based responses in Mg^2+^-free solution were profoundly suppressed by the selective N-methyl-D-aspartate (NMDA) receptor antagonist D-AP5. In addition, application of NMDA to the retina produced excitatory inward currents in DACs. These data strongly suggest that DACs express functional NMDA receptors. We further found that in the presence of Mg^2+^, D-AP5 reduced the peak amplitude of melanopsin-based DAC responses by ~70% when the cells were held at their resting membrane potential (−50 mV), indicating that NMDA receptors are likely to contribute to retrograde signal transmission to DACs under physiological conditions. Moreover, our data show that melanopsin-based NMDA-receptor-mediated responses in DACs are suppressed by antagonists specific to either the NR2A or NR2B receptor subtype. Immunohistochemical results show that NR2A and NR2B subunits are expressed on DAC somata and processes. These results suggest that DACs express functional NMDA receptors containing both NR2A and NR2B subunits. Collectively, our data reveal that, along with AMPA receptors, NR2A- and NR2B-containing NMDA receptors mediate retrograde signal transmission from ganglion cell photoreceptors to DACs.

## Introduction

Light can be converted into electrical signals by rod and cone photoreceptors. These signals are transmitted, via bipolar cells, to ganglion cells that project directly to brain nuclei related to image-forming vision. Light can also be transduced into electrical signals by a small population of retinal ganglion cells expressing the photopigment melanopsin; this opsin is encoded by *Opn4* (Provencio et al., 1998; Berson et al., 2002; Hattar et al., 2002). The axons of these intrinsically photosensitive retinal ganglion cells (ipRGCs) project primarily to brain nuclei related to non-image-forming visual functions (Hattar et al., 2003; Lucas et al., 2003). Before leaving the retina, however, the primary axons of some, if not all ipRGCs, bifurcate and form axon collaterals projecting back toward the outer retina (Joo et al., 2013). These collaterals likely make excitatory synapses with a subclass of amacrine cells, dopaminergic amacrine cells (DACs), forming a retrograde signaling pathway within the retina (Zhang et al., 2008, 2012; Atkinson et al., 2013; Dkhissi-Benyahya et al., 2013; Newkirk et al., 2013; Prigge et al., 2016; Zhao et al., 2017). Given that dopamine acts on almost all retinal neurons, reconfiguring retinal electrical and chemical synapses (Lasater, 1987; McMahon et al., 1989; Knapp et al., 1990; Mills et al., 2007), ipRGCs likely influence rod and cone pattern vision via the dopaminergic system (Allen et al., 2014; Prigge et al., 2016).

In the retrograde neural pathway, ipRGCs appear to utilize action potentials to activate N-type Ca^2+^ channels, allowing an influx of Ca^2+^ into the axon collateral terminal and thereby triggering glutamate release onto DACs (Prigge et al., 2016). Glutamate receptors can be classified into two groups: metabotropic and ionotropic glutamate receptors. Three types of ionotropic glutamate receptors have been identified: N-methyl-D-aspartate (NMDA) receptors, α-amino-3-hydroxyl-5-methyl-4-isoxazole-propionate (AMPA) receptors, and kainate receptors. We have previously demonstrated that AMPA receptors contribute to retrograde signal transmission to DACs, with a very minor or non-existent contribution from kainate receptors (Zhang et al., 2007, 2012). However, NMDA-receptor-mediated responses have not been carefully examined on isolated or intact DACs (Gustincich et al., 1997; Zhang et al., 2007, 2012). The extracellular solution used in previous studies normally contained Mg^2+^, which blocks NMDA channels in a voltage-dependent fashion (Mayer et al., 1984). Extracellular Mg^2+^ could eliminate NMDA-receptor-mediated signal transmission (if it exists). Therefore, it remains unclear whether NMDA receptors contribute to retrograde signal transmission to DACs.

A functional NMDA receptor consists of two obligatory NR1 subunits and two NR2 subunits, of which there are four subtypes, NR2A, NR2B, NR2C and NR2D (Ishii et al., 1993; Monyer et al., 1994; Laube et al., 1998; Dingledine et al., 1999). DACs were recently found to express NR1 subunits (Fasoli et al., 2017), suggesting that NMDA receptors could play a role in signal transmission to DACs. The NR2 subunits, which have not yet been confirmed to be expressed on DACs, are thought to confer distinct physiological and pharmacological properties on the NMDA receptor (Monyer et al., 1992; Flint et al., 1997; Chen et al., 1999; Tovar and Westbrook, 1999; Sagdullaev et al., 2006).

In this study, we utilized immunohistochemical, electrophysiological, pharmacological and genetic methods to investigate the postsynaptic mechanisms responsible for retrograde excitatory signal transmission. Our results suggest that NR2A- and NR2B-containing NMDA receptors mediate retrograde signal transmission to DACs along with AMPA receptors.

## Materials and Methods

### Animals

Adult male and female mice were used for all experiments. The animals were housed in the Oakland University animal facility on a 12:12-h light-dark cycle, with lights on at 07.30 h. Food and water were available *ad libitum*. All procedures conformed to NIH guidelines for laboratory animals and were approved by the Institutional Animal Care and Use Committee at Oakland University.

The *tyrosine hydroxylase (TH)* driven red fluorescent protein (RFP) mouse line (originally generated on a C57BL/6J background at Vanderbilt University; Zhang et al., 2004) was imported to Oakland University. *TH*::RFP mice were crossed with triple-knockout mice (BL6/129) in which the cone-photoreceptor-specific cyclic nucleotide channel *Cnga3*, rod-specific-G protein transducin α-subunit *Gnat1* and *Opn4* had been deleted (Hattar et al., 2003). From multiple crossings, *TH*::RFP transgenic mice homozygous for the *Gnat1* and *Cnga3* mutations (*Cnga3*^−/−^ Gnat1^−/−^ TH::RFP) were bred on a mixed C57BL/129 background (henceforth, we refer this mouse line as “*Opn4*-only *TH*::RFP”). *TH*::RFP transgenic mice bred on a mixed C57BL/129 background with wild-type *Gnat1*, *Cnga3* and *Opn4* genes were also used (referred to as wild-type *TH*::RFP mice). Two to 4-month-old *Opn4*-only and wild-type *TH*::RFP mice were used for the present study. In addition, *retinal degeneration 1* (*rd1*) mice homozygous for the *Pde6b*^rd1^ mutation were originally purchased from The Jackson Laboratory. In *rd1* mice, rod loss occurs rapidly, with onset at postnatal day 8 (P8) and nearly complete loss by P21. By P90, virtually all outer photoreceptors have been lost except for ~3% of cone somata in the dorsal retina (Carter-Dawson et al., 1978). We crossed this line with our *TH*::RFP transgenic line to obtain *rd1 TH*::RFP transgenic mice. This mouse line carried a mixed C57BL/6J and C3H background. *rd1 TH*::RFP mice used in this study were between 4 and 8 months old.

### Electrophysiology Recording

All experiments used a flat-mount retina preparation and were conducted during the day to avoid a circadian effect. Mice were dark adapted for 1–2 h before experiments, then euthanized by CO_2_ overdose and cervical dislocation. Eyes were enucleated under infrared illumination and transferred to a petri dish filled with oxygenated extracellular solution containing (in mM) 125 NaCl, 2.5 KCl, 1 MgSO_4_, 2 CaCl_2_, 1.25 NaHPO_4_, 20 glucose and 26 NaHCO_3_. Under a dim red light, the cornea and lens were removed from the eyes, and the retina was separated from the sclera. The retina was then placed with the photoreceptor side down in a recording chamber mounted on the stage of an upright conventional fluorescence microscope (Axio Examiner, Zeiss, Oberkochen, Germany). Oxygenated extracellular medium (pH 7.4, bubbled with 95% O_2_-5% CO_2_) continuously perfused the recording chamber at a rate of 2–3 ml/min, and was maintained at 32~34°C by a temperature control unit (TC-344B, Warner Instruments, Hamden, CT, USA).

The isolated retina was maintained in darkness for approximately 1 h prior to recording. Cells and recording pipettes were viewed on a computer monitor coupled to a digital camera (AxioCam, Zeiss, Oberkochen, Germany) mounted on the microscope. After being visualized by fluorescence using a rhodamine filter set, *TH*::RFP-expressing cells were randomly selected for recording. The identified cells and glass electrodes were then visualized using infrared differential interference contrast (IR-DIC) optics. Experiments began 10–15 min after the cells were located using fluorescence, which allowed the retina to recover from photobleaching caused by the fluorescence excitation light. Recovery may have been incomplete during this short dark-adaptation period, so our experiments were likely performed in a partially light-adapted state.

Whole-cell voltage-clamp recordings were made from the soma of RFP-labeled DACs using 7–10 MΩ electrodes and an Axopatch 200B amplifier (Molecular Devices, Sunnyvale, CA, USA). The intracellular solution for the whole-cell voltage-clamp experiments contained (in mM) 120 Cs-methane sulfonate, 5 EGTA, 10 HEPES, 5 CsCl, 5 NaCl, 0.5 CaCl_2_, 4 Na-ATP, 0.3 Na-GTP and 5 lidocaine n-ethyl-chloride (QX-314); the pH was adjusted to 7.2–7.4 with CsOH. QX-314 was used to block intrinsic Na^+^-channel-mediated action potentials in DACs, thus highlighting extrinsic light-induced inward currents in the cells and improving the space clamp quality of the voltage clamp. The liquid junction potential was measured to be −10 mV and was corrected. All electrophysiological data were acquired using a Digidata 1440A digitizer (Molecular Devices, Sunnyvale, CA, USA).

D-2-amino-5-phosphonopentanoate (D-AP5) and L-(+)-2-amino-4-phosphonobutyric acid (L-AP4) were purchased from Hello Bio (Avonmouth, UK). All other chemicals were obtained from Tocris Bioscience (Ellisville, MO, USA). The drugs were stored in frozen stock solutions and dissolved in intracellular or extracellular solution before experiments.

### Light Stimulation

Light stimuli were generated using a 470-nm LED (LED Supply, Randolph, VT, USA; and LC Corp, Brooklyn, NY, USA) to stimulate the melanopsin chromophore (peak sensitivity of approximately 480 nm). An LED controller (Mightex, Pleasanton, CA, USA) was used to drive the LED; the light intensity was adjusted by varying the driving current. The light intensity was measured at the surface of the retina using an optical power meter (units converted from μW/cm^2^ to photons·cm^−2^·s^−1^; model 843-R, Newport, Irvine, CA, USA). A light intensity of 4.7 × 10^13^ photons·cm^−2^·s^−1^ was used for all experiments.

**Figure 1:**
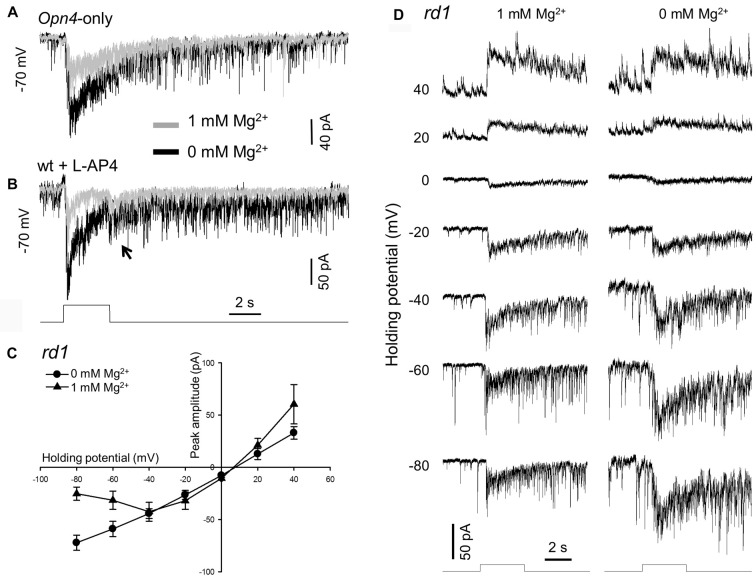
Removing extracellular Mg^2+^ increases melanopsin-based dopaminergic amacrine cell (DAC) responses. Melanopsin-based DAC responses were recorded using a whole-cell voltage-clamp technique in flat-mount retinae isolated from *Opn4*-only **(A)**, wild-type **(B)** or *rd1* **(C,D)** tyrosine hydroxylase (TH)::red fluorescent protein (RFP) mice. **(A)** The peak amplitude of a melanopsin-based DAC response recorded in the presence of 1 mM Mg^2+^ (gray trace) was smaller than that recorded in the absence of Mg^2+^ (black trace). **(B)** Results similar to those observed in **(A)** were also obtained in a wild-type *TH*::RFP retina with rod and cone input blocked by 50 μM L-AP4. Gray trace: 1 mM Mg^2+^; black trace: 0 mM Mg^2+^. The transient inward current observed at light cessation is highlighted (arrow). **(C)** Current-voltage (I-V) curves constructed from 4 DACs recorded in *rd1* *TH*::RFP retinae with (triangles) and without (circles) 1 mM Mg^2+^. A cocktail of inhibitory antagonists was used in this experiment, which included 20 μM GABAzine, 50 μM TPMPA and 1 μM strychnine. **(D)** Representative recordings from one of the DACs used to construct the I-V curves in **(C)**. Stimulation bar shows timing of 470 nm light pulse (duration: 3 s; intensity: 4.7 × 10^13^ photons·cm^−2^·s^−1^).

### Immunohistochemistry

Wild-type mice were euthanized by asphyxiation with CO_2_, followed by cervical dislocation. One eye was enucleated from each mouse and rapidly dissected in oxygenated extracellular solution. Eyecups were fixed for 15 min in 4% paraformaldehyde in 0.1 M PBS, then rinsed briefly in 0.1 M PBS, transferred to 30% sucrose, and incubated overnight at 4°C. Eyecups were frozen in a sucrose/OCT solution and cut into 12 μm sections using a cryostat (Leica CM3050 S, Wetzlar, Germany) for vertical retinal immunostaining. For whole-mount immunostaining, retinae were dissected from freshly enucleated eyes in oxygenated PBS. Retinae were fixed for 15 min in 4% paraformaldehyde in 0.1 M PBS.

Retina slices and wholemount retinae were blocked for 2 h with 1% BSA (Fisher Scientific, Hampton, NH, USA) and 0.3% Triton X-100 (Sigma-Aldrich Corp., St. Louis, MO, USA) in 0.1 M PBS. A primary antibody against NR2A (rabbit polyclonal, concentration 1:500, AB1555P, EMD Millipore, Billerica, MA, USA) or NR2B (rabbit polyclonal, concentration 1:200, AB1557P, EMD Millipore, Billerica, MA, USA), was applied with an antibody against TH (mouse monoclonal, concentration 1:1000, MAB318, EMD Millipore, Billerica, MA, USA) and incubated overnight for retina slices. Wholemount retinae were incubated with two primary antibodies for two overnight periods. Following incubation in primary antibody, samples were rinsed in 0.1 M PBS and incubated in appropriate secondary antibodies (donkey anti-rabbit Alexa 488 and donkey anti-mouse Alexa 594, or donkey anti-mouse Alexa 488 and donkey anti-sheep Alexa 594; concentration 1:500; Life Technologies, Carlsbad, CA, USA) for 2 h. Samples were rinsed again in 0.1 M PBS before being coverslipped with Vectashield Hard-Set mounting solution (Vector Laboratories, Burlingame, CA, USA).

### Confocal Imaging and Analysis

Retina slices were visualized with a confocal microscope (TCS SP8, Leica Microsystems, Wetzlar, Germany) at 63× magnification, using sequential scanning to eliminate crosstalk between fluorophores. All images were collected as z-stacks with 0.36 μm spacing. Dendrite images were deconvolved using NIS Elements AR. To illustrate co-localization, a single slice was selected from each image stack. Fiji was used to adjust the brightness and contrast of each channel for clarity (Schindelin et al., 2012, 2015). A merged image was then created, and points of co-localization were noted.

Wholemount retinae were visualized using a Nikon Eclipse Ti confocal microscope (Nikon Instruments, Tokyo, Japan) at 60× magnification. Sequential scanning was used to eliminate crosstalk between fluorophores. Images of DAC somata were collected as z-stacks with a spacing of 0.5 μm; images of DAC dendrites were also collected as z-stacks, but with a spacing of 0.3 μm.

### Data Analyses

Electrophysiological data were analyzed using the Clampfit 10.4 (Molecular Devices, Sunnyvale, CA, USA) and SigmaPlot 12.0 (Systat Software, Germany) software packages. The light response was measured as the peak current evoked by light onset. To construct current-voltage curves, the peak current amplitude at each holding potential for each cell was normalized by dividing by the maximum peak current for that cell. Normalized peak currents from different cells at the same holding potential were averaged and then plotted against the holding potential. To assess the effects of pharmacological agents, the reduction of the light-induced peak current amplitude was evaluated for significance using a paired *t*-test. Values are presented as the mean ± SEM. *p* < 0.05 was considered statistically significant.

**Figure 2:**
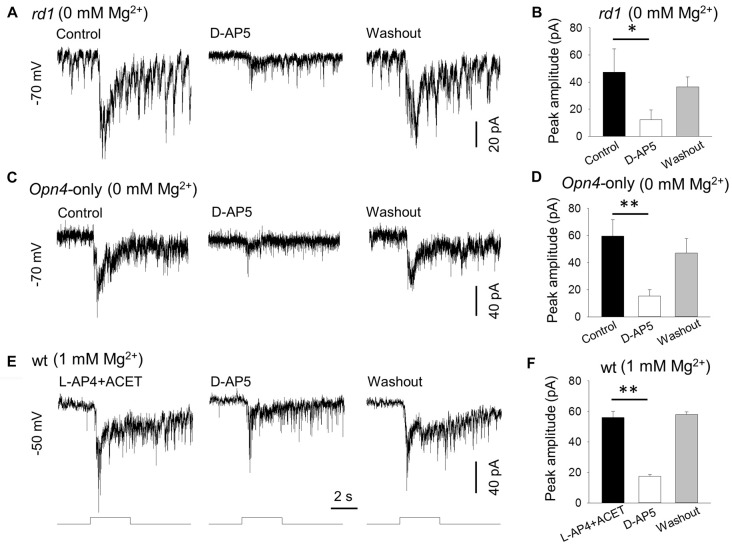
D-AP5 suppresses melanopsin-based DAC responses with and without extracellular Mg^2+^. In Mg^2+^-free solution, 50 μM D-AP5 significantly suppressed melanopsin-based responses of DACs recorded in *rd1* *TH*::RFP retinae in the presence of the inhibitory cocktail as described in Figure 1 (**A,B**, *p < 0.05, *n* = 4) and in *Opn4*-only *TH*::RFP retinae (**C,D**, **p < 0.01, *n* = 6). All cells were held at −70 mV. To mimic physiological conditions, DACs were recorded in wild-type *TH*::RFP retinae and held at −50 mV (close to the resting membrane potential) in 1 mM Mg^2+^ solution. L-AP4 and ACET were used to block rod and cone input to DACs. Under these conditions, the melanopsin-based responses were significantly suppressed by D-AP5 (**E,F**, **p < 0.01, *n* = 4). Stimulation bar shows timing of 470 nm light pulse (duration: 3 s; intensity: 4.7 × 10^13^ photons·cm^−2^·s^−1^).

## Results

### Removing Mg^2+^ from Extracellular Solution Enhances Melanopsin-Based DAC Responses

As stated above, our previous studies were performed with extracellular Mg^2+^ that could block NMDA receptors (if any) on DACs (Zhang et al., 2007, 2012). To clarify whether DACs express functional NMDA receptors, we re-examined melanopsin-based DAC responses with and without extracellular Mg^2+^. Melanopsin-based DAC responses were examined using three strategies in the present study, with each method possessing advantages and disadvantages. The first strategy was to use *Opn4*-only *TH*::RFP mice to isolate melanopsin-based responses in DACs. Since Mg^2+^ suppresses NMDA-receptor-mediated currents when the membrane potential is more negative than −40 mV (Mayer et al., 1984), we recorded DAC light-induced responses at a holding potential of −70 mV with and without Mg^2+^. A 470-nm light flash with a duration of 3 s and an intensity of 4.7 × 10^13^ photons·cm^−2^·s^−1^ was delivered to the retina every 2 min to evoke DAC responses. Figure 1A illustrates light-induced responses of a DAC with (gray trace) and without 1 mM Mg^2+^ (black trace). In the presence of Mg^2+^, an inward current was observed at light increment (referred to as an ON response), which was desensitized during the stimulus. After light cessation, the desensitized current persisted for over 15 s before returning to the baseline, accompanied by relatively high-frequency excitatory postsynaptic events. The dynamic characteristics of the response observed in the absence of Mg^2+^ were similar to those noted in the presence of Mg^2+^ (Figure 1A, black trace). However, the peak amplitude of the response was remarkably increased (Figure 1A, black trace). Average data showed that removing Mg^2+^ increased the peak current from 64.0 ± 14.3 pA to 169.0 ± 41.2 pA (*n* = 6, *p* < 0.05).

Although the gross anatomy of the retina is largely unaffected by the deletion of rod and cone function in *Opn4*-only *TH*::RFP mice, there is minor degeneration with age (Calvert et al., 2000). To rule out the possibility that the Mg^2+^-mediated inhibition of melanopsin-based DAC responses is a result of retinal degeneration, we developed a second approach. Using wild-type *TH*::RFP retinae, we repeated the experiments described above in the presence of L-AP4, an agonist of mGluR6. L-AP4 was used to block rod- and cone-driven excitatory responses to DACs through ON bipolar cells (Zhang et al., 2007, 2008). Consistent with our previous report (Prigge et al., 2016), L-AP4-resistant ON DAC responses were observed in wild-type *TH*::RFP retinae. Figure 1B illustrates an example (gray trace). When Mg^2+^ was removed from the extracellular solution, the peak current of the ON response was markedly increased (Figure 1B, black trace). Similar results were obtained in three additional cells. Furthermore, a response was observed at light decrement (referred to as an OFF response, Figure 1B, arrow). Since this OFF response has previously been reported to be mediated by inhibitory amacrine cells driven by OFF bipolar cells (Qiao et al., 2016), we did not further analyze the effect of Mg^2+^ on this response.

To determine whether Mg^2+^ suppresses melanopsin-based DAC responses in a voltage-dependent manner, we measured the current-voltage relationships of melanopsin-based DAC responses with and without Mg^2+^. To completely isolate direct melanopsin input from ipRGCs, we developed a third approach using *rd1* *TH*::RFP mice. We included a cocktail of inhibitory blockers (20 μM GABAzine—a GABA_A_ receptor antagonist; 50 μM TPMPA—a GABA_C_ receptor antagonist; and 1 μM strychnine—a glycine receptor antagonist) to block possible inhibitory input from amacrine cells driven by ipRGCs (Reifler et al., 2015). Melanopsin-based responses were measured with and without 1 mM Mg^2+^ at voltages ranging from −80 mV to +40 mV in steps of 20 mV. Four cells were used to construct each current-voltage curve (Figure 1C); recordings from a representative cell are shown in Figure 1D. It was found that both current-voltage curves showed a similar reversal potential, near 0 mV (the excitatory ion reversal potential; Figure 1C). However, while the current-voltage curve measured in the absence of Mg^2+^ was nearly linear, the current-voltage curve measured in the presence of Mg^2+^ appeared to be made up of two components—a region of negative slope and region of positive slope (Figure 1C). The region of negative slope from −80 mV to −40 mV reflects the voltage-dependent Mg^2+^ blockade of NMDA receptors.

### D-AP5 Suppresses Melanopsin-Based DAC Light Responses

To further confirm the expression of NMDA receptors on DACs, we examined the effect of D-AP5 (a selective NMDA receptor antagonist) on the melanopsin-based DAC response in Mg^2+^-free solution. We first tested 50 μM D-AP5 on DACs recorded from *rd1* *TH*::RFP retinae in the presence of the inhibitory cocktail described above, and found that application of DAP-5 resulted in a suppression of melanopsin-based DAC responses (Figure 2A). Pooled data showed that D-AP5 reduced melanopsin-based DAC responses by 78% (Figure 2B, 47.3 ± 17.2 pA vs. 12.3 ± 7.3 pA, *p* < 0.05, *n* = 4). Similar results were obtained from DACs recorded in *Opn4*-only *TH*::RFP retinae in the absence of the inhibitory cocktail (Figure 2C). On average, D-AP5 suppressed melanopsin-based DAC responses by 74% (Figure 2D, 59.5 ± 12.1 pA vs. 15.3 ± 4.6 pA, *p* < 0.01, *n* = 6). These results suggest that, in Mg^2+^-free solution, NMDA receptors are responsible for approximately 75% of the melanopsin-based DAC response, whereas AMPA and kainate receptors mediate about 25% of the response.

To determine whether D-AP5 attenuates melanopsin-based DACs responses under physiological conditions, we examined the effect of D-AP5 on the responses of wild-type DACs in the presence of 1 mM Mg^2+^. We used L-AP4 (50 μM) and ACET (0.3 μM) to block excitatory and inhibitory input, respectively, from classical photoreceptors (Qiao et al., 2016). Under these conditions, we found that D-AP5 reduced the peak amplitude of melanopsin-based DAC responses by 69% (Figures 2E,F, 56.0 ± 4.1 pA vs. 17.5 ± 1.2 pA, *p* < 0.01, *n* = 4) at a holding potential of −50 mV (this potential is close to the DAC resting membrane potential; Newkirk et al., 2013). This result suggests that, at a physiological concentration of Mg^2+^, NMDA receptors mediate 69% of the melanopsin-based DAC response, with the remaining 31% of the response mediated by AMPA receptors.

### Exogenous NMDA Induces an Inward Current in DACs

**Figure 3:**
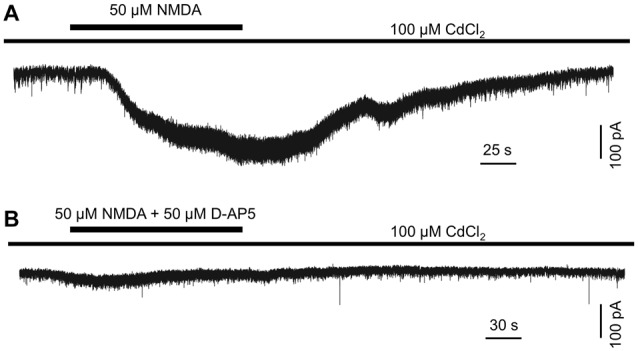
N-methyl-D-aspartate (NMDA) induces an excitatory inward current in DACs. Drug application is indicated by horizontal line above current traces. Synaptic inputs to the DAC were blocked by 100 μM Cd^2+^. **(A)** 50 μM NMDA (with 2 μM NMDA receptor co-agonist glycine; 1 μM strychnine was used to block currents induced by the action of glycine on glycine receptors) induced an inward current in a DAC. **(B)** In the presence of 50 μM D-AP5, almost no current was induced by NMDA/glycine.

The above data suggest that endogenous glutamate activates NMDA receptors, which serve to mediate DAC light responses. If this is the case, exogenous NMDA should evoke an excitatory current in DACs. To test this hypothesis, we used Cd^2+^ (100 μM) to block any excitatory or inhibitory input to DACs in wild-type *TH*::RFP retinae. A solution containing NMDA (50 μM) and the NMDA receptor co-agonist glycine (2 μM) was used to induce NMDA-receptor-mediated currents. Since glycine could activate glycine receptors on DACs, the glycine receptor antagonist strychnine (1 μM) was added to the extracellular solution. As shown in Figure 3A, the combination of 50 μM NMDA and 2 μM glycine induced an inward current of 239 pA in a DAC held at −70 mV in Mg^2+^-free solution. Similar results were obtained in four other cells. In addition, the NMDA-induced current was almost completely eliminated in the presence of 50 μM D-AP5 (Figure 3B; *n* = 5), suggesting that NMDA specifically acts on DAC NMDA receptors.

**Figure 4:**
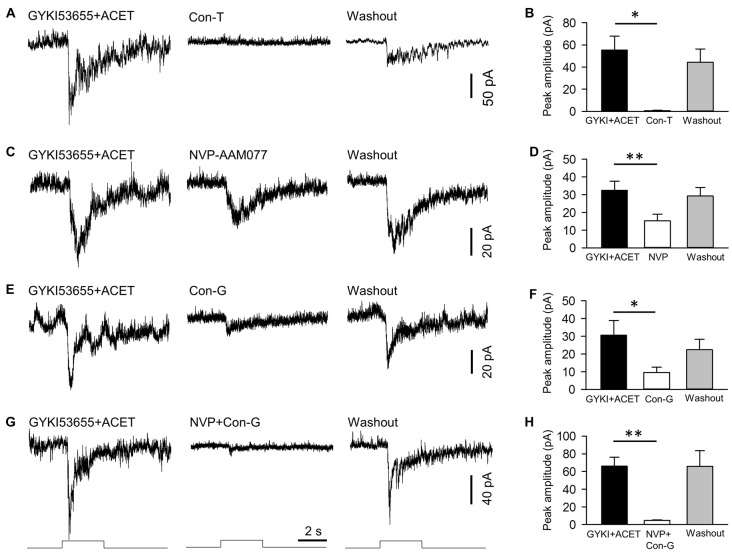
NMDA receptors of DACs contain NR2A and NR2B subtypes. NMDA-receptor-mediated melanopsin-based DAC currents were recorded in *Opn4*-only *TH*::RFP retinae with α-amino-3-hydroxyl-5-methyl-4-isoxazole-propionate (AMPA) and kainate receptors blocked by GYKI53655 and ACET, respectively. DACs were held at −40 mV, at which potential Mg^2+^ does not block NMDA-receptor-mediated currents. The NMDA-receptor-mediated response was completely blocked by 20 μM conantokin-T (Con-T), an NMDA receptor antagonist specific for receptors containing NR2 subunits (**A,B**, *p < 0.05, *n* = 5). 0.1 μM NVP-AMM077 (NVP), an NMDA receptor antagonist specific for the NR2A subunit, significantly suppressed the DAC response (**C,D**, **p < 0.01, *n* = 4). In addition, 0.3 μM conantokin-G (Con-G), an NMDA receptor antagonist specific for the NR2B subunit, significantly reduced DAC responses (**E,F**, *p < 0.05, *n* = 5). Finally, co-application of NVP and Con-G almost completely eliminated DAC responses (**G,H**, **p < 0.01, *n* = 5). Stimulation bar shows timing of 470 nm light pulse (duration: 3 s; intensity: 4.7 × 10^13^ photons·cm^−2^·s^−1^).

### NR2A, NR2B and NR2A/NR2B Antagonists Attenuate NMDA-Receptor-Mediated Melanopsin-Based DAC Responses

We next determined the particular subtypes of NMDA receptors expressed on DACs. As shown in Figure 1C, Mg^2+^ had no effect on melanopsin-based DAC responses at a holding potential of −40 mV. Therefore, we recorded melanopsin-based DAC responses in *Opn4*-only *TH*::RFP retinae at a holding potential of −40 mV in the presence of 1 mM Mg^2+^. To isolate currents mediated by NMDA receptors, the AMPA receptor antagonist GYKI53655 (30 μM) and kainate receptor antagonist ACET (0.3 μM) were applied to the retina. In the presence of these antagonists, melanopsin-based DAC responses were completely blocked by D-AP5 (data not shown, *n* = 5), suggesting that the currents not blocked by GYKI53655 and ACET were mediated exclusively by NMDA receptors. Isolating NMDA-mediated currents allowed us to determine the relative contributions of the various NR2 subunits. To examine the role of the NR2 subunit in signal transmission, we evaluated the effect of synthetic conantokin-T (Con-T), a small γ-carboxyglutamate-containing peptide that specifically blocks NR2-containing NMDA receptors (Klein et al., 2001; Huang et al., 2014). We found that Con-T (20 μM) eliminated melanopsin-based DAC responses (Figures 4A,B).

**Figure 5:**
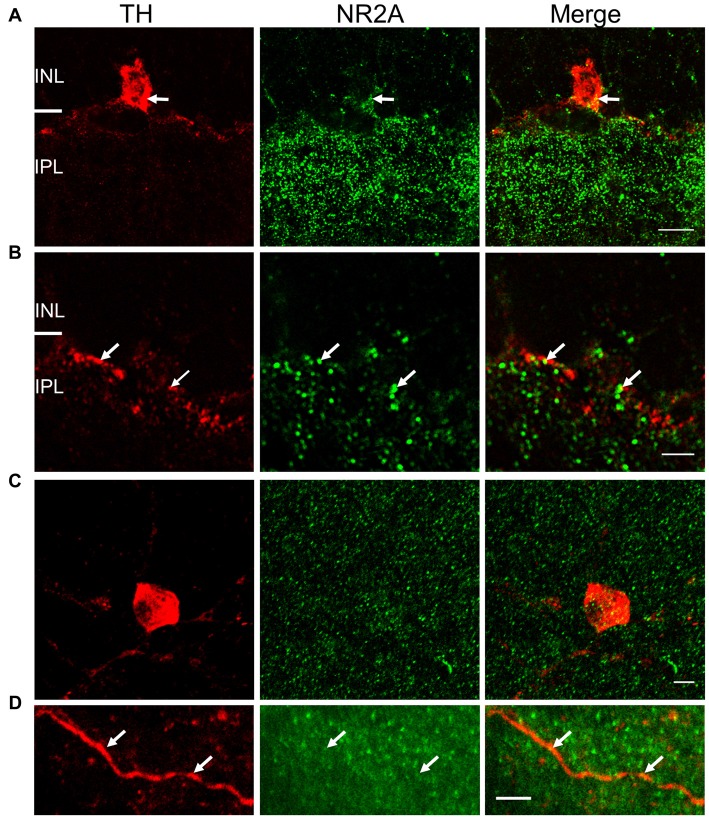
NR2A subunit is expressed on TH-positive somata and processes. For **(A)** and **(B)**, immunostaining was performed in vertical cryostat sections of the retina. Each row displays a single optical section (0.36 μm), viewed in different color channels. **(A)** Left panel (red): TH-labeled soma of a cell and processes; middle panel (green): intense punctate immunofluorescence of NR2A in the inner plexiform layer (IPL) and sparse distribution of punctate labeling in the inner nuclear layer (INL); right panel: merge showing co-localization of TH and NR2A on TH-labeled soma and processes (yellow), especially at arrows pointing to identical positions in the soma. **(B)** Arrows point to co-localization (yellow, right panel) of TH (red, left panel) and NR2A (green, middle panel) on DAC processes. Scale bar, 10 μm for **(A)** and 3 μm for **(B)**. For **(C,D)**, immunostaining was performed in wholemount retinae. **(C)** Maximum projection of a Z-stack illustrating co-localization of NR2A (green) and TH (red). **(D)** Single image of a DAC dendrite illustrating sparse co-localization of NR2A and TH (arrow). Scale bars for **(C**,**D)** are 5 μm.

To determine the contribution of the NR2A subunit, we applied the selective NR2A antagonist NVP-AMM077. We found that this antagonist (0.1 μM) reduced the peak amplitude of the inward current by 54% (Figures 4C,D, 32.5 ± 5.1 pA vs. 15.3 ± 3.8 pA, *p* < 0.01, *n* = 4). This result suggests that at least 54% of functional NMDA receptors contain NR2A subunits. To determine the contribution of the NR2B subunit, we tested the effect of the selective NR2B antagonists conantokin-G (Con-G) and ifenprodil. We found that Con-G (0.3 μM) reduced the peak current amplitude by 67% (Figures 4E,F, 30.6 ± 8.3 pA vs. 9.6 ± 2.9 pA, *p* < 0.05, *n* = 5). A similar effect was observed with ifenprodil (data not shown). These results suggest that at least 67% of functional NMDA receptors contain NR2B subunits. When NVP-AMM077 (0.1 μM) and Con-G (0.3 μM) were co-applied, the peak current amplitude of the responses was reduced by 92% (Figures 4G,H, 66.0 ± 10.3 pA vs. 4.6 ± 0.6 pA, *p* < 0.01, *n* = 5). This result further confirms that functional DAC NMDA receptors contain either NR2A or NR2B subunits, or a mixture of both.

### NR2A and NR2B Immunostaining on DACs

To validate the physiological evidence for the expression of NMDA receptors on DACs, we performed immunohistochemical studies in wild-type mice to label both DACs and either the NR2A or NR2B subunit of the NMDA receptor. In vertical retinal slices, the NR2A antibody exhibited dense punctate labeling in the IPL (Figure 5A, middle panel). Punctate labeling was observed on cell bodies in the inner nuclear layer (INL); some of these cell bodies were also labeled by the TH antibody (Figure 5A), suggesting that the NR2A subunit is present on the somata of DACs. Putative co-localization of the NR2A subunit with TH was also observed on DAC dendrites (Figure 5B). To quantify these putative co-localizations, we performed whole-mount retinal immunostaining using antibodies against NR2A and TH. We found that all cell bodies labeled by the TH antibody also expressed NR2A (Figure 5C). On average, 15.7 ± 2.4 putative co-localizations of TH and NR2A were found per soma (*n* = 11). Co-localizations were also observed on DAC dendrites (Figure 5D). Average of co-localizations was 1.1 ± 0.4 per 10 μm dendrite.

**Figure 6:**
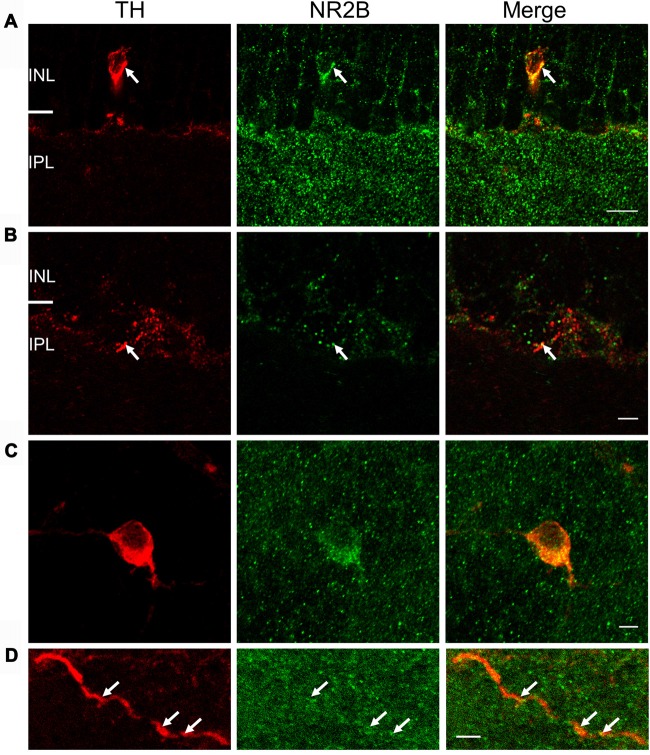
NR2B subunit is expressed on TH-positive soma and processes. For **(A)** and **(B)**, immunostaining was performed in vertical cryostat sections of the retina. Each row displays a single optical section (0.36 μm), viewed in different color channels. **(A)** Left panel (red): TH-labeled soma and processes of a cell; middle panel (green): strong punctate immunofluorescence of NR2B in the inner plexiform layer (IPL) and sparse distribution of punctate labeling in the INL right panel: merge showing co-localization of TH and NR2B on TH-labeled soma and processes (yellow), especially at arrows pointing to identical positions in the soma. **(B)** Arrows point to co-localization (yellow, right panel) of TH (red, left panel) and NR2B (green, middle panel) on a DAC process. Scale bar, 10 μm for **(A)** and 3 μm for **(B)**. For **(C,D)**, immunostaining was performed in wholemount retinae. **(C)** Maximum projection of a Z-stack illustrating co-localization of NR2B (green) and TH (red). **(D)** Single image of a DAC dendrite illustrating co-localization of NR2B and TH (arrow). Scale bars for **(C,D)** are 5 μm.

Co-localization of NR2B and TH immunostaining was also observed in vertical retinal slices (Figures 6A,B) and whole-mounts (Figures 6C,D). In vertical retinal slices, the NR2B antibody displayed dense punctate staining throughout the IPL as well as labeling cells in the INL (Figure 6A). Some of these cells were immunoreactive for the TH antibody (Figure 6A), suggesting that DAC somata express the NR2B subunit. Examination of TH-positive dendrites also revealed putative co-localization of TH and NR2B (Figure 6B). In whole-mount retinae, averages of putative co-localizations of TH and the NR2B subunit on DAC somata (Figure 6C) and dendrites (Figure 6D) were 43 ± 3.9 per soma (*n* = 10) and 1.9 ± 0.4 per 10 μm dendrite, respectively. In combination, these data demonstrate that the NMDA receptor subunits NR2A and NR2B are both expressed on DACs.

## Discussion

We have previously demonstrated that AMPA receptors mediate intra-retinal retrograde signaling from ipRGCs to DACs (Zhang et al., 2012). The present study has expanded upon our previous work by revealing that NMDA receptors are also involved in retrograde melanopsin signaling. We further demonstrated that functional NMDA receptors in DACs contain NR2A and NR2B subunits. These results provide new information about the postsynaptic mechanisms underlying retrograde signal transmission in the retina. In conjunction with our previous work (Zhang et al., 2008; Prigge et al., 2016), we now have a clearer picture of the origin (M1 ipRGCs), route (axon collateral), presynaptic mechanism (action potential-dependent N-type calcium channel), postsynaptic mechanism (AMPA and NMDA receptors) and physiological function (facilitation of cone-mediated light adaptation) of this novel retrograde melanopsin signaling pathway.

### DACs Express Functional NMDA Receptors

NMDA receptors have been shown to contribute to synaptic signal transmission and plasticity in the CNS (Collingridge and Lester, 1989). In the vertebrate retina, NMDA receptors and their functions have been relatively well characterized in retinal ganglion cells (Coleman and Miller, 1988; Zhou et al., 1994) (Chen and Diamond, 2002; Zhang and Diamond, 2009; Poleg-Polsky and Diamond, 2016). So far, however, reports of whether amacrine cells express functional NMDA receptors are inconsistent. Although studies have demonstrated the existence of NMDA receptors on amacrine cells (Slaughter and Miller, 1983; Massey and Miller, 1990; Hartveit and Veruki, 1997; Dumitrescu et al., 2006; Zhou et al., 2016), there are reports showing that NMDA receptors contribute very little, if at all, to amacrine cell light responses (Lukasiewicz and McReynolds, 1985; Coleman and Miller, 1988; Boos et al., 1993; Tran et al., 1999). In the present study, our results provide solid evidence showing that functional NMDA receptors are expressed in the DAC, an important amacrine cell subtype that releases the neurotransmitters GABA and dopamine (Hirasawa et al., 2015). This evidence includes the observation that removing extracellular Mg^2+^ profoundly enhanced melanopsin-based DAC responses at a holding potential of −70 mV in all three animal models (wild-type, *Opn4*-only and *rd1* mice) used. In addition, the current-voltage relationship constructed from melanopsin-based DAC responses recorded in *rd1* retinae in the presence of Mg^2+^ had a “J-shape”, while the telltale region of negative slope was absent when Mg^2+^ was removed from the extracellular solution. This suggests that DACs express functional NMDA receptors, as reported previously in other neurons (Mayer et al., 1984; Nowak et al., 1984; Cohen, 2000; Manookin et al., 2010).

However, our data demonstrated that Mg^2+^ suppressed NMDA receptors at membrane potentials more negative than −40 mV, which is a more negative potential than that reported for other neurons (Mayer et al., 1984; Nowak et al., 1984). There are several possible explanations for this phenomenon. First, expression of NR3A could reduce the sensitivity of NMDA receptors to Mg^2+^ (Zhou et al., 2009, 2015); however, this is not likely to be the case for DACs, as the data show that DAC NMDA receptors are primarily composed of NR1 and NR2 subunits (see discussion below). Secondly, reducing the concentration of Mg^2+^ may also enhance the response of other inhibitory amacrine cells that express NMDA receptors and inhibit DACs, thus reducing the sensitivity of DAC NMDA receptors to Mg^2+^. This is also unlikely, as inhibitory inputs to DACs were blocked with GABA and glycine receptor antagonists. A third explanation takes into account the fact that signal transmission from ipRGCs to DACs is primarily mediated by presynaptic voltage-gated Ca^2+^ channels on ipRGC axon terminals (Prigge et al., 2016). Removing extracellular Mg^2+^ could increase the negative charge on the extracellular surface of ipRGC axon terminals (Cadetti et al., 2004), inducing a negative shift in the activation of voltage-gated Ca^2+^ channels. This negative shift of Ca^2+^ channel activation may result in decreased signal transmission from ipRGCs to DACs. As a result, the current-voltage curve of melanopsin-based DAC responses in Mg^2+^-free solution could shift leftward for negative holding potentials and rightward for positive holding potentials (Figure 1C). Such a leftward shift could offset Mg^2+^-free-solution-induced potentiation of NMDA-receptor-mediated currents, resulting in no change in the amplitude of the currents between 0 mV and −40 mV in Mg^2+^-free solution.

The increased melanopsin-based DAC responses observed in Mg^2+^-free solution were substantially suppressed by D-AP5, providing further evidence for the presence of functional NMDA receptors in DACs. Additionally, in the presence of Cd^2+^ (which blocks all synaptic input to DACs), exogenous NMDA induced excitatory currents in DACs. Since these NMDA-induced currents were blocked by D-AP5, it is most likely that they were induced by the action of NMDA on DAC NMDA receptors. Collectively, our biophysical and pharmacological data strongly suggest that DACs express functional NMDA receptors which mediate glutamatergic input from ipRGCs to DACs.

Our results also suggest that NMDA receptors could be involved in mediating rod and cone input to DACs, as these cells receive input from classical photoreceptors as well as from ipRGCs (Zhang et al., 2007, 2008; Newkirk et al., 2013; Zhao et al., 2017). However, for several reasons, we did not attempt to investigate whether NMDA receptor antagonists have an effect on rod/cone-mediated DAC responses. One reason is that rod signals reach DACs through the primary rod pathway, which consists of rod bipolar cells and AII amacrine cells (Zhao et al., 2017). It is likely that the expression of NMDA receptors on AII amacrine cells would make the results of such a study ambiguous (Hartveit and Veruki, 1997; Zhou et al., 2016). In addition, cones can excite DACs through ON cone bipolar cells, both directly and indirectly (via ipRGCs). The expression of NMDA receptors on ipRGCs could also interfere with the interpretation of data (Chen and Diamond, 2002; Jakobs et al., 2007). In contrast, signal transmission from ipRGCs to DACs appears to operate across a single synapse (Zhang et al., 2008; Prigge et al., 2016), which provides an ideal model system to study DAC NMDA receptors.

### Functional DAC NMDA Receptors Contain NR2A and NR2B Subunits

Two obligatory NR1 subunits and two NR2 subunits (of which there are four subtypes, NR2A-D) make up a functional NMDA receptor (Ishii et al., 1993; Monyer et al., 1994; Laube et al., 1998; Dingledine et al., 1999). Con-T has been demonstrated to be a non-competitive antagonist for NMDA receptors containing NR2 subunits in transferred Xenopus oocytes, cultured hippocampal neurons, and retinal ganglion cells (Klein et al., 1999, 2001; Huang et al., 2014). Our data show that, similar to D-AP5, Con-T abolishes NMDA-receptor-mediated DAC responses, suggesting that DAC NMDA receptors contain NR2 subunits. Indeed, both NR2A- and NR2B-specific antagonists partially blocked NMDA-receptor-mediated responses in DACs, and co-application of these antagonists almost completely eliminated the response. Although we cannot rule out the involvement of NR2C and NR2D subunits, our physiological data suggest that NR2A and NR2B are the primary components of the functional NMDA receptors expressed on DACs. In addition, NR2B subunits are likely to contribute more than NR2A subunits, as the suppression of NMDA-receptor-mediated currents by the NR2B antagonist (67%) was greater than that by the NR2A antagonist (54%).

These conclusions are also supported by immunohistochemical data obtained in the current study and previous publications. The expression of NMDA receptor subunits has been investigated mostly by *in situ* hybridization and immunohistochemistry in rat retinae (Brandstätter et al., 1994; Kreutz et al., 1998; Fletcher et al., 2000; Kalloniatis et al., 2004; Zhang and Diamond, 2006, 2009). NR1 and NR2A-D subunits are expressed in virtually all cells in the ganglion cell layer and in subsets of amacrine cells in the INL (Brandstätter et al., 1994; Kreutz et al., 1998; Fletcher et al., 2000; Kalloniatis et al., 2004; Zhang and Diamond, 2006, 2009). Limited studies in the mouse retina show that NR2A and NR2B subunits are expressed in the inner retina; however, the cellular localization is unclear (Watanabe et al., 1994; Gustafson et al., 2013; Namekata et al., 2013). Our data reveal that both the NR2A and NR2B subunits are expressed in TH-positive somata and processes. Combined with a previous study in the mouse retina showing that DACs express NR1 immunoreactivity (Fasoli et al., 2017), it appears that a functional DAC NMDA receptor contains NR1/NR2A, NR1/NR2B or NR1/NR2A/NR2B. The greater expression of NR2B subunits (compared to NR2A subunits) on DAC somata and dendrites (Figures 5, 6) further supports the hypothesis that NR2B subunits may contribute to functional DAC NMDA receptors more than NR2A subunits.

NMDA receptors are expressed in both synaptic and extrasynaptic membranes. The specific membrane localization may be determined by the NR2 subtype of the receptor (Stocca and Vicini, 1998; Rumbaugh and Vicini, 1999; Momiyama, 2000). In the rat retina, NR2A-containing receptors are located at synaptic sites, while NR2B-containing receptors are present in extrasynaptic sites (Hartveit et al., 1994; Zhang and Diamond, 2009). If this is also the case in the mouse retina, our data would suggest that DAC NMDA receptors are confined to synaptic and extrasynaptic sites. Further studies are needed to test this hypothesis.

### Possible Roles of NMDA Receptors in the Physiology and Pathophysiology of DACs

Previous work has demonstrated that ipRGCs are likely to signal to DACs, facilitating cone-mediated light adaptation via dopamine signaling (Prigge et al., 2016). During this process, the presynaptic release of glutamate from ipRGCs activates DAC AMPA receptors, which mediate fast excitatory synaptic signal transmission (Zhang et al., 2007, 2012). The presence of NMDA receptors on DACs (as demonstrated in this study) likely complements AMPA-mediated synaptic transmission. Newkirk et al. (2013) have reported that the resting membrane potential of DACs is approximately −50 mV. We found that NMDA receptors mediate ~70% of DAC light responses at this potential in the presence of Mg^2+^. These results suggest that, under physiological conditions, NMDA receptors contribute to signal transmission to DACs to a greater extent than AMPA receptors. In addition, AMPA receptors have relatively fast kinetic characteristics, whereas NMDA receptors have slower kinetic characteristics. The presence of NMDA receptors could increase the duration of synaptic currents in DACs. Finally, NMDA receptors have high Ca^2+^ permeability, resulting in an influx of Ca^2+^ into DACs during signal transmission. This increase in intracellular Ca^2+^ could lead to long-term changes in synaptic strength and other cellular modifications such as alterations in synaptic connectivity. Future studies may determine whether NMDA receptors mediate the plasticity of DACs during light adaptation through these mechanisms.

In addition to their normal physiological roles, NMDA receptors also contribute to excitotoxicity in the CNS (Collingridge and Lester, 1989). This NMDA-receptor-mediated excitotoxicity leads to prolonged Ca^2+^ influx, which results in neuronal injury and death. Loss of DACs has been reported in animal models of diabetic retinopathy and retinopathy of prematurity (Gastinger et al., 2006; Aung et al., 2014; Spix et al., 2016); however, the mechanisms involved are unknown. Although exploring the mechanisms of cell death in these conditions is beyond the scope of the present study, our results imply that the vulnerability of DACs in neurodegenerative diseases may be due, at least partially, to NMDA-receptor-mediated excitotoxicity. If this is the case, the NR2A and NR2B antagonists (such as Con-T and Con-G) tested in the present study could be potentially used to control or prevent DAC excitotoxicity in neurodegenerative diseases of the retina.

## Author Contributions

L-LL designed the study, conducted patch-clamp experiments, analyzed patch-clamp data, and contributed to the writing of the article. NJS conducted immunohistochemical experiments, performed image analysis, and contributed to the writing of the article. D-QZ designed the study and wrote the article. All authors approved the final version of the manuscript.

## Conflict of Interest Statement

The authors declare that the research was conducted in the absence of any commercial or financial relationships that could be construed as a potential conflict of interest.
